# Fe_3_O_4_@PDA@PEI Core-Shell Microspheres as a Novel Magnetic Sorbent for the Rapid and Broad-Spectrum Separation of Bacteria in Liquid Phase

**DOI:** 10.3390/ma15062039

**Published:** 2022-03-10

**Authors:** Yueqi Zhang, Bin Du, Yuting Wu, Zhiwei Liu, Jiang Wang, Jianjie Xu, Zhaoyang Tong, Xihui Mu, Bing Liu

**Affiliations:** State Key Laboratory of NBC Protection for Civilian, Beijing 102205, China; qi.qguai@163.com (Y.Z.); dubin51979@163.com (B.D.); wuyuting9585@163.com (Y.W.); liuzhw07@126.com (Z.L.); roverman@163.com (J.W.); juechen1981@163.com (J.X.); billzytong@126.com (Z.T.); muxh0511@163.com (X.M.)

**Keywords:** magnetic beads, bacterial enrichment, magnetic separation, electrostatic interaction

## Abstract

Bacterial infection is a significant cause of morbidity and mortality to humans worldwide. Thus, a method for nonspecific, sensitive, and rapid enrichment of such bacteria is essential for bacteria detection and treatment. This study demonstrates a self-made core-shell Fe_3_O_4_@Polydopamine@Polyethyleneimine magnetic beads (Fe_3_O_4_@PDA@PEI MBs) with a high density positive charge-based magnetic separation scheme for the broad-spectrum rapid enrichment of microorganisms in the liquid phase. MBs with a high-density positive charge have a strong electrostatic attraction to most microorganisms in nature. Our scheme is as follows: (1) wrapping dopamine (DA) on the iron oxide through self-polymerization and wrapping PEI on the outermost shell layer in a mode of crosslinking with the PDA; (2) subsequently, the Fe_3_O_4_@PDA@PEI MBs were used to concentrate microorganisms from the sample solution; (3) performing magnetic separation and calculating the adsorption efficiency. The as-prepared Fe_3_O_4_@PDA@PEI MBs composite was carefully characterized by zeta potential analysis, Value stream-mapping (VSM), transmission electron microscopy (TEM), and Fourier transforms infrared spectrometry (FT-IR). In this study, both gram-positive and gram-negative bacteria could be captured in three minutes through electrostatic interaction. Furthermore, the adsorption efficiency on gram-negative (>98%) is higher than that on gram-positive (>95%), allowing for a simple, rapid assay to enrich organisms in resource-limited settings.

## 1. Introduction

Severe diseases caused by a bacterial infection or microbial contamination pose a severe threat to the public health status and are considered one of the most significant existing problems worldwide [1,2,3]. The World Health Organization (WHO) estimated that food contaminated with parasites, bacteria, viruses, toxins, or chemicals have affected more than 600 million people worldwide with 420,000 deaths, causing about $ 95 billion in productivity losses in low- and middle-income economies annually [4]. Nowadays, a consensus has been reached that if the reproduction and transfer of bacteria can be detected at a low level and then detected in a timely and effective manner, the reproduction and transfer of bacteria can be inhibited in force [5,6,7]. Moreover, it is also of great significance to realize the rapid and effective detection of bacteria in maintaining world biosafety and biological anti-terrorism. The research in this field is also in the ascendant. The rapid detection of bio-harmful bacteria is also helpful in preventing and treating diseases. In developing countries, microbial contamination is a critical health issue [8]. In consequence, in the fields of environment, food, clinical, quality management, and so on, it is an urgent matter to develop a low-cost, convenient, rapid, accurate, and bacteria-rich screening approach.

Low concentrations of bacteria in the environment can cause people to suffer from possible diseases, but these concentrations are often far beyond the detection range of instruments. Previous microbiology methods, including bacterial culture [9] or multiplex polymerase chain reaction (PCR) assay [10,11], immunology-based methods [12,13], have the following problems: long detection time, vulnerability to microbial contamination, limited laboratories of professional inspection institutions, and the inability to enrich and detect bacteria on-site in time [14]. Therefore, efficient enrichment of bacteria is a necessary and urgent matter, and the commonly used nanocomposite for electrostatic adsorption of bacteria are polyethersulfone nanofibrous membranes [15], flexible graphene oxide (GO) microsheets [16], and so on, but the production conditions of these materials are harsh, and the production process is complex.

Iron oxide is a widely used magnetic material due to its excellent biocompatibility under certain conditions [17]. Iron oxide can be surface modified according to different functions and applications [18,19,20]. It has crucial applications in the cell, protein, nucleic acid, and microorganism separation. Due to its obvious advantages of lower production cost, good efficiency and simplified operation, modified iron oxide provide a convenient method for rapid enrichment of bacteria, which is considered as a powerful means to improve early clinical intervention, food safety regulation, and environment surveillance [21,22,23]. 

In previous studies, it was found that functional groups (carboxylates, aldehydes, and amines, etc.) could be modified on iron oxide, and then biologically active substances (such as antigens, antibodies) outside the functional groups could be used in various directions [24]. Indeed, the biological affinity modification of iron oxide improves its specificity, but at the same time, its ability to enrich microorganisms without discrimination is also reduced. Some studies have shown that if iron oxide is chemically modified with chitosan [25], amino [26], vancomycin [27], or unmodified [28,29], it can be used for non-specific separation of a variety of bacteria. This non-specific separation is mainly driven by the electrostatic force between the bacteria with a negative charge and the magnetic beads with positive charge [30]. Increasing electrostatic interaction is conducive to improving the enrichment efficiency of iron oxide on bacteria.

DA is an attractive, functional monomer, which can self-polymerize under alkaline conditions to form a robust hydrophilic PDA membrane on various materials [31]. Iron oxide coated with PDA has outstanding adhesion and excellent biocompatibility, and the surface can be further modified due to its rich carboxyl and amino groups [32]. PEI is a polymer with the highest cationic density in existing materials. PEI is usually composed of linear and branched forms, which contain rich amino and imine. The abundant N atoms on the macromolecular chain of PEI make it highly proton-philic. When pH < 10, the amine groups on the molecular chain are mostly protonated [33]. Therefore, PEI can be called a cationic polyelectrolyte, which can enrich most of the bacteria in nature through electrostatic interaction. PEI has potential application prospects in sample pretreatment. 

Therefore, our team synthesized a novel Fe_3_O_4_@PDA@PEI magnetic beads (Fe_3_O_4_@PDA@PEI MBs) complex. The advantages of self-polymerization of DA are used to reduce the manufacture craft of iron oxide. Furthermore, PEI at the outermost layer is currently known as a polymer with the highest positive charge density so that it can be adsorbed on the negatively charged bacteria near the neutral pH by strong electrostatic adsorption. Due to the rich cations and excellent adsorption capacity of PEI, a new adsorbent Fe_3_O_4_@PDA@PEI MBs with satisfactory broad-spectrum enrichment efficiency was prepared, then enriched with the bacteria in the liquid phase. Finally, we calculated the capture efficiency (CE) using the cultured-based method. 

Adenosine triphosphate (ATP) is existing in all viable cells, and the ATP content in each cell remains relatively stable (−10^−18^ mol/cell) [34]. Therefore, the determination of ATP is a determinant of bacterial cell concentration. Firefly bioluminescence is usually used to detect ATP because the number of photons (BL intensity) is proportional to ATP concentration. ATP can be detected indirectly by firefly luciferase-ATP-BL reaction. The capture efficiency of Fe_3_O_4_@PDA@PEI MBs on bacteria can be validated by ATP-BL method in addition to the culture-based method. 

Given the situation mentioned above, it is possible to optimize the sensitivity of the ATP-BL assay by combining the advantages of PDA, PEI, and magnetic nanoparticles. The preparation condition of Fe_3_O_4_@PDA@PEI MBs is mild, and it can be operated at room temperature. Fe_3_O_4_@PDA@PEI MBs can achieve on-site rapid and broad-spectrum enrichment in large industries, such as medical, quarantine, environmental, agricultural, pharmaceutical, and food processing.

## 2. Materials and Methods

### 2.1. Materials and Chemicals

Fe_3_O_4_ was supplied by Promega Ltd. Dopamine hydrochloride was purchased from Aladdin Chemistry Co. Ltd. (Shanghai, China). Polyethyleneimine (PEI, M.W. 600,000–1,000,000, 50%) was bought from Sigma-Aldrich Co. (Shanghai, China). Deionized (DI) water (18.2 MΩ cm) was purified by a Smart-Q deionized water system (Hitech Pure Water Technology, Shanghai, China) and used in all aqueous solutions. Luria-Bertani (LB) medium was from Beijing Land Bridge Technology Co., Ltd. (Beijing, China).

Scanning electron microscopy (SEM) was conducted via UHR FE-SEM SU8020 (Hitachi, Japan). The morphologies of the as-prepared materials were indicated by transmission electron microscopy (TEM, FEI Tecnai G2 F30, Hillsboro, OR, USA). Infrared analysis was conducted with a Nicolet IS50 Fourier transform infrared FT-IR (Thermofisher, Waltham, MA, USA). The zeta-potential was determined by the Malvern Nano Z Zetasizer. Bacteria were cultured by the RTS-1 Fully Automatic Biological Growth Monitoring Reactor (Republic of Latvia). ATP bioluminescence was detected by Lumitester Smart (Kikkoman Biochemifa Co., Noda, Japan). *Escherichia coli* (*E. coli* ATCC8739) and *Bacillus subtilis* (ATCC6633) were purchased from Guangdong culture Collectioncenter of Microbiology.

### 2.2. Preparation of Bacteria Samples

*E. coli* and *Bacillus subtilis* were selected and cultured in 30 mL LB medium at 37 °C and 900 rpm for continuous oscillation. The rough concentration of bacteria in the broth was derived by measuring the absorbance at a wavelength of 850 nm using RTS-1 Fully Automatic Biological Growth Monitoring Reactor. The culture was grown to the mid-log phase (OD_600_ = ~0.6). After centrifugation at 6000 g for 10 min, the supernatant was removed and resuspended with 0.85% NaCl at pH 7.4 and repeated three times. The bacterial solution was then continuously diluted to the desired concentration for subsequent use. A total of 100 μL bacterial solution of each accurate concentration was coated on a LB agar plate to confirm the viable count (CFU·mL^−1^). All containers were sterilized in a 120 °C high-pressure sterilizer for 30 min before and after use.

### 2.3. Synthesis and Characterization of Fe_3_O_4_@PDA@PEI Magnetic Beads (MBs)

**Figure 1:**
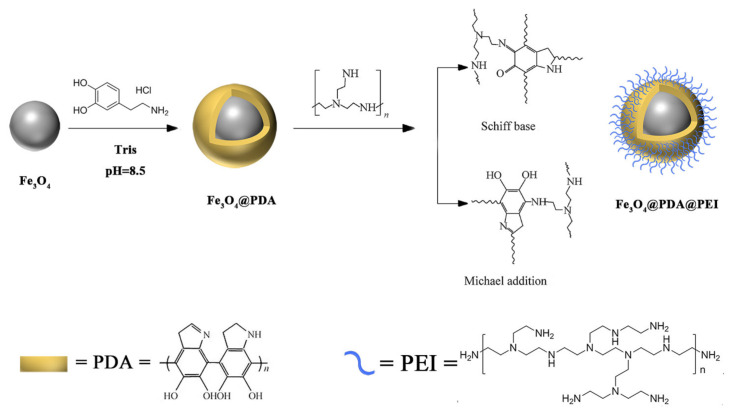
Schematic diagram of Fe_3_O_4_@PDA@PEI MBs preparation process.

Based on previously reported procedures [23], Fe_3_O_4_ NPs were modified by PDA as follows (Figure 1): 10 mg of the Fe_3_O_4_ MBs (2 mL, 5 mg·mL^−1^) was firstly dispersed in 10 mL of Tris-HCl buffer (10 mM, pH 8.5). Then, 10 mL watery solution inhering 7 mg DA was added to the suspension and get with properly intensity agitate continuously for 7 h at normal atmospheric temperature. Afterwards, Fe_3_O_4_@PDA MBs were collected by a magnet, washed with deionized water 5 times, washed with ethanol 3 times, and finally dispersed in water (30 mL).

Secondly, Fe_3_O_4_@PDA@PEI MBs were synthesized (Figure 1). The Fe_3_O_4_@PDA MBs in water buffer were added to PEI solution (25% 30 mL) and agitated for 10 h at room temperature. The Fe_3_O_4_@PDA MBs were cleaned up at least three times with water and ethanol to remove excess PEI. Under the action of the external magnetic field, the prepared Fe_3_O_4_@PDA@PEI MBs were separated from the reaction solvent and washed several times with deionized water and ethanol. Finally, the Fe_3_O_4_@PDA@PEI MBs were stored in water (1 mg·mL^−1^) at 4 °C for further use. The derived offspring was flushed out with deionized water several times and desiccated in a vacuum. The prepared samples (Fe_3_O_4_, Fe_3_O_4_@PDA@PEI MBs) were distributed in H_2_O and observed by TEM. The samples were in geometry of transmission used for IR investigation. The prepared solid MBs were mixed with KBr powder for tableting and then placed on the sample holder for observation. The zeta potentials were measured for the samples with a concentration of 0.025 mg·mL^−1^. PBS was used as a titrand for testing and the initial concentration was 0.01 mol·L^−1^. Fe_3_O_4_ and Fe_3_O_4_@PDA@PEI were detected in the temperature range of 2–400 k for VSM.

### 2.4. Enrichment of E. coli and Bacillus Subtilis Using Fe_3_O_4_@PDA@PEI MBs

**Figure 2:**
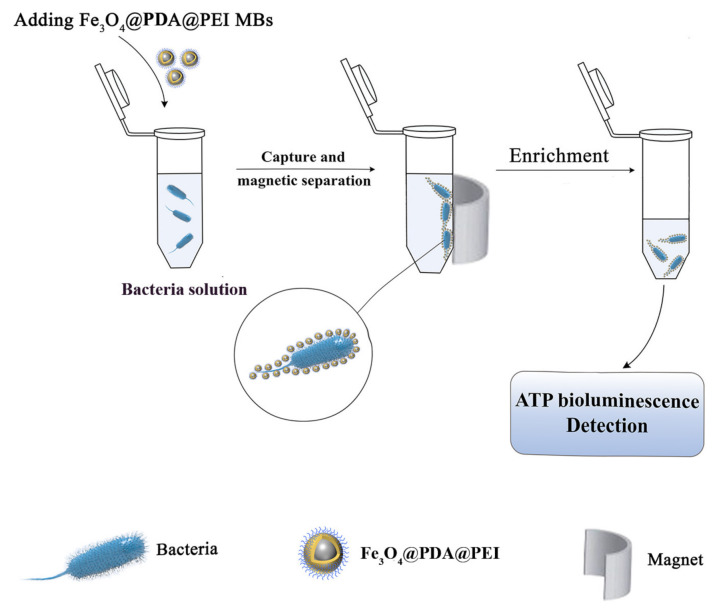
Schematic representation of enrichment and detection of bacteria using Fe_3_O_4_@PDA@PEI MBs.

The enrichment process takes place in two steps, capturing and enriching *E. coli* and *Bacillus subtilis* to form the MBs-bacterial conjugate shown in Figure 2.

Firstly, the bacterial solution was diluted to a certain level with normal saline (0.85% *w*/*v*). Plate counting and ATP-BL determination were performed to determine the concentration of the bacterial solution. Then take 1 mL bacterial suspension, add 1 mL Fe_3_O_4_@PDA@PEI MBs (1 mg·mL^−1^), mix well, and culture at room temperature. After the separation of MBs- bacterial conjugates with external magnets, the supernatant was added to the coating plate and counted. After magnetic separation, 50 μL supernatant was taken for ATP-BL detection. The MBs-bacterial conjugate was washed twice with normal saline and separated for subsequent use.

Finally, the pre-enriched bacteria solution and the enriched supernatant solution are coated on the nutrient solution. Fe_3_O_4_@PDA@PEI-*Bacillus subtilis* conjugates and Fe_3_O_4_@PDA@PEI-*E.coli* conjugates were preserved with 2.5% glutaraldehyde and analyzed by SEM.

### 2.5. ATP-BL Detection

The BL experimental procedure consisted in 50 μL bacteria solution that was pipetted into the cotton swab of the LuciPac Pen. Then, the swab was returned into the swab holder of the LuciPac Pen. The end of the swab holder was pushed into the luciferase–luciferin mixture solution stored in the LuciPac Pen, and the powdered reagent was shaken well to dissolve thoroughly. The LuciPac Pen was inserted to measure the cleanliness indication by inserting into the measurement chamber of Lumitester PD-30 with readings presented as relative light units (RLU). Amounts of bacteria in sample dilutions were confirmed as colony forming units (CFU) from triplicate plates.

## 3. Results

### 3.1. Characterization of the Composite

#### 3.1.1. TEM and VSM Analysis

**Figure 3:**
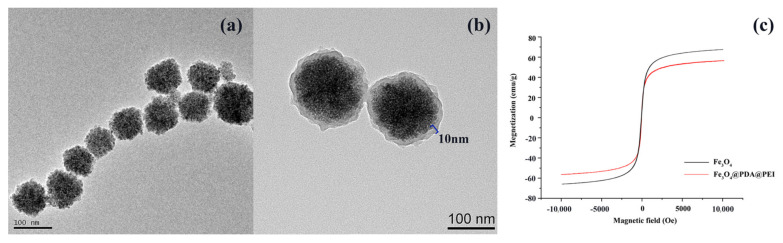
TEM images of (**a**) Fe_3_O_4_ and (**b**) Fe_3_O_4_@PDA@PEI; (**c**) VSM images of Fe_3_O_4_ and Fe_3_O_4_@PDA@PEI in the temperature of 300 k.

As shown in Figure 3a, Fe_3_O_4_ NPs are uniform highly monodisperse nanomagnetic beads with a diameter of 100 nm. The middle PDA layer and positively charged PEI shell assembled into a bright ultrathin shell (10 nm) in the periphery of the Fe_3_O_4_ core (Figure 3b), for further electrostatic adherence of negatively charged bacteria. In practical applications, magnetic materials must possess enough magnetism to guarantee rapid separation from liquid [35]. Therefore, we tested the magnetism of Fe_3_O_4_@PDA@PEI MBs at room temperature by vibrating the sample magnetometer (Figure 3c). It can be seen that the whole magnetic hysteresis loops are approximatively S-shaped, and the coercivity and remanence are equal to zero, demonstrating that the prepared Fe_3_O_4_@PDA@PEI MBs were super-paramagnetic. The saturation magnetization of Fe_3_O_4_@PDA@PEI MBs was 58.7 emu/g.

#### 3.1.2. Size and Zeta Potential Analysis

**Figure 4:**
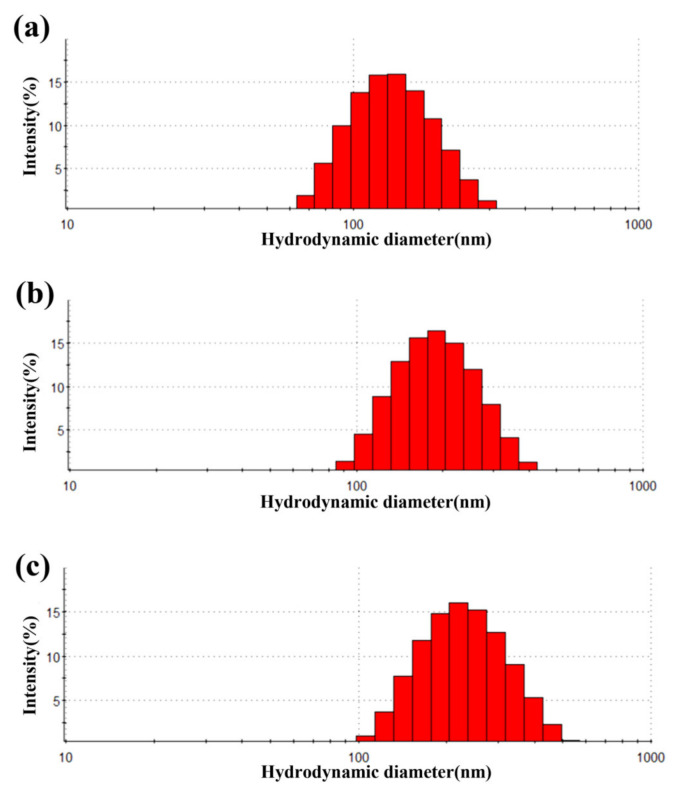
Variation of hydrodynamic diameter. (**a**) Hydrodynamic diameter of Fe_3_O_4_, (**b**) Fe_3_O_4_@PDA, and (**c**) Fe_3_O_4_@PDA@PEI MBs. (pH = 7.4, 25 °C, 0.025 mg/mL).

It can be seen from Figure 4 that with the layer-by-layer coating of PDA and PEI, the hydrodynamic diameter of the magnetic beads has increased significantly. Controlling the mass ratio of DA and Fe_3_O_4_ and the DA coating time to control the thickness of the intermediate layer to regulate the particle size of the entire magnetic beads is the key to the preparation process. The experimental results show that the mass ratio of DA and Fe_3_O_4_ is 1:7 when Fe_3_O_4_ is coated with PDA, and the final formed magnetic beads have the highest cationic density. The suitable coating time of PDA is 20–25 min.

**Figure 5:**
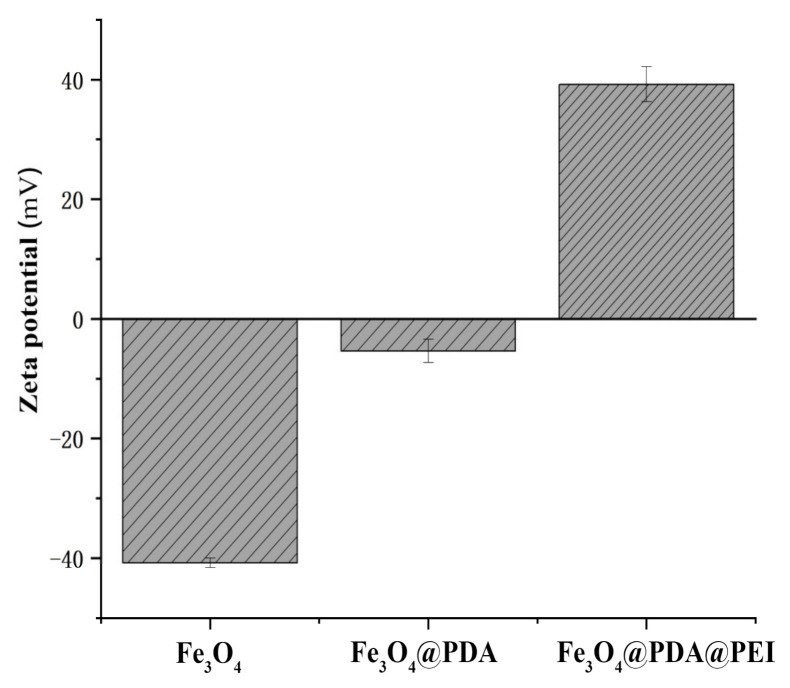
Zeta potential changes. (pH = 7.4, 25 °C, 0.025 mg/mL).

Figure 5 shows the Zeta potential changes of Fe_3_O_4_, Fe_3_O_4_@PDA, and Fe_3_O_4_@PDA@PEI MBs. After modification, the absolute value of the Zeta potential of Fe_3_O_4_@PDA@PEI MBs increased, indicating that the dispersion of the system improved. Because the modification of PEI on the surface of Fe_3_O_4_ reduces the surface energy of nanoparticles, inhibits the agglomeration of particles to a certain extent, and improves the dispersion of the system. Figure 5 indicates the Zeta potential of the unmodified Fe_3_O_4_ is negative, while that of the modified Fe_3_O_4_@PDA@PEI MBs is positive, which is due to the carboxyl and other groups with an electronegative charge on the surface of Fe_3_O_4_. Due to the coating of PEI with high positive charge density on the surface of Fe_3_O_4_, the surface of Fe_3_O_4_@PDA@PEI MBs have a large number of amino groups with positive charge [36], indicating that Fe_3_O_4_@PDA@PEI MBs were successfully prepared.

#### 3.1.3. FT-IR Analysis

**Figure 6:**
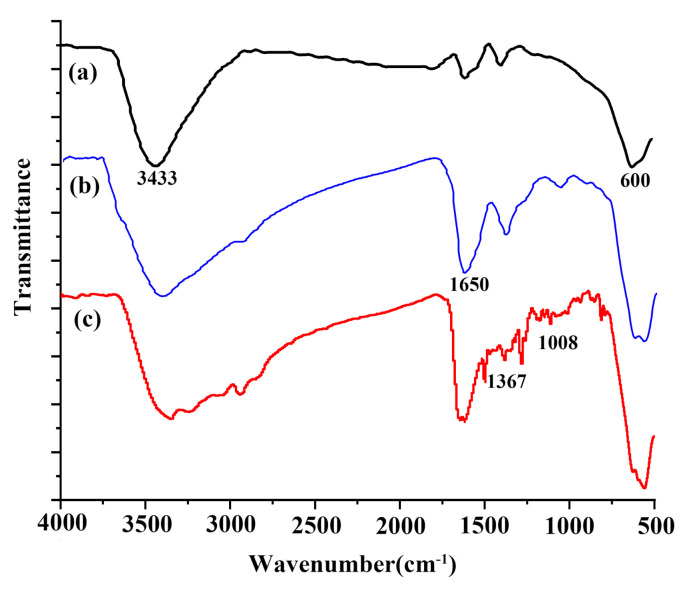
Infrared spectra of (**a**) Fe_3_O_4_, (**b**) Fe_3_O_4_@PDA and (**c**) Fe_3_O_4_@PDA@PEI MBs.

As shown in Figure 6a–c (The transmittance is not absolute, but the curve has been moved to highlight the measurement highlights.), the three spectra have strong absorption peaks at 600 cm^−1^, corresponding to the vibration peak of the Fe-O bond of Fe_3_O_4_ magnetic nanoparticles [37]. Figure 6b shows strong absorption peaks at 1650–1450 cm^−1^ and 1050 cm^−1^ in which the characteristic absorption peak of PDA corresponds to the deformation vibration of N-H and the stretching vibration of aromatic C-N, respectively [38]. Figure 6b proves that PDA forms on the surface of Fe_3_O_4_ nanoparticles. In Figure 6c, a strong absorption peak was observed at 1650–1600 cm^−1^, corresponding to the Michael addition between PDA and PEI and the C=N and C=O structures formed by Schiff base reaction [39,40]. A strong absorption peak was observed at 3000–2800 cm^−1^ and 1450 cm^−1^, belonging to the stretching vibration peak of -CH_2_- in PEI and the in-plane bending vibration peak, respectively. The wide absorption band at 2980–3670 cm^−1^ corresponded to the stretching vibration of -NH- and -OH in PEI [41]. Above all, PEI modification was successful.

#### 3.1.4. Isoelectric Point Determination of Fe_3_O_4_@PDA@PEI

**Figure 7:**
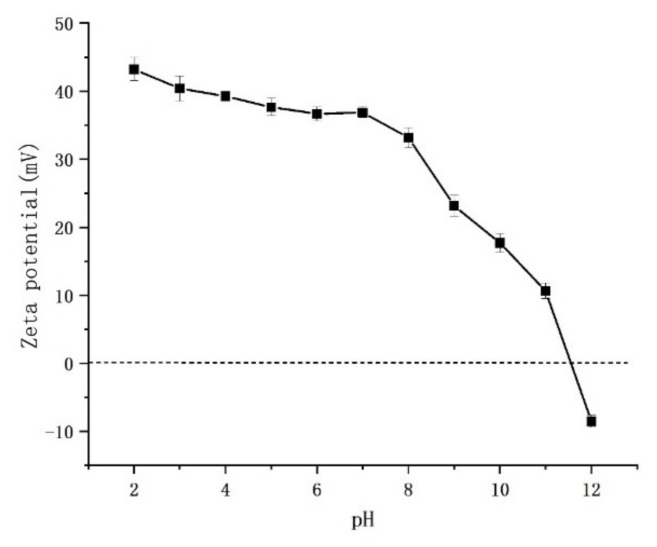
Isoelectric point of Fe_3_O_4_@PDA@PEI.

For sake of ulteriorly proving the Fe_3_O_4_@PDA@PEI MBs were fabricated successfully, the zeta potentiometer of Fe_3_O_4_@PDA@PEI was measured in different pH solutions varying from 2 to 12. The surface adsorption potential of MBs was characterized by Zeta potential analyzer. As shown in Figure 7, the Zeta potential of PEI-modified magnetic fluid was positive and decreased with the increase of pH value. When the pH value is less than the isoelectric point, it is a positive charge; otherwise, it is negative. By the positive surface potential of the magnetic fluid under alkaline conditions, it can be determined that the cationic polymer PEI has been well bonded to the surface of iron oxide particles. The decrease of Zeta potential with the increase of pH value is determined by the hydrolysis of PEI:

[-CH_2_CH_2_NH-] + H_2_O→[-CH_2_CH_2_NH_2_-]^+^ + OH. (1)

As the pH of the solution increases, the reaction proceeds, and the surface potential of the modified particles decreases. The conjugation of PEI makes Fe_3_O_4_@PDA@PEI MBs carry positive charges in the wide pH range of 2–11.

These results demonstrate that PEI can be successfully used to modify Fe_3_O_4_ NPs. The modification of PEI will change the particle size, particle size distribution, and charged properties of Fe_3_O_4_ NPs. The successful preparation of Fe_3_O_4_@PDA@PEI MBs laid a foundation for exploring the interaction between magnetic nanoparticles and bacteria.

### 3.2. Interaction between Surface-Modified Fe_3_O_4_@PDA@PEI MBs and Bacteria

#### 3.2.1. SEM Analysis

**Figure 8:**
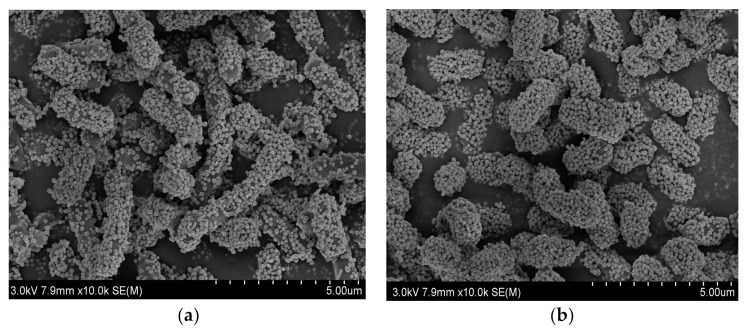
SEM images of (**a**) Fe_3_O_4_@PDA@PEI MBs-*Bacillus subtilis* conjugates and (**b**) Fe_3_O_4_@PDA@PEI MBs-*E. coli* conjugates.

The SEM images of Fe_3_O_4_@PDA@PEI-*Bacillus subtilis* conjugates and Fe_3_O_4_@PDA@PEI-*E.coli* conjugates are shown in Figure 8, which confirmed the effective capture of *Bacillus subtilis* and *E. coli* by Fe_3_O_4_@PDA@PEI MBs.

#### 3.2.2. The Influence of the Amount of Fe_3_O_4_@PDA@PEI MBs and the Action Time on the Binding Effect

Michael J. measured the surface charge density of bacteria by the saturation of adsorbed ions, and the results showed that the negative charge density of the lipopolysaccharide coated outer surface of Gram-negative bacteria (6.6 ± 1.3 nm^−2^) was deduced to be seven times larger than that of the protein surface layer of Gram-positive bacteria (1.0 ± 0.2 nm^−2^) [42]. In this article, *Bacillus subtilis* and *E. coli* were chosen to represent respectively Gram-positive and Gram-negative bacteria. 

Under near-neutral conditions, the Zeta potentials of Gram-negative bacteria and Gram-positive bacteria represented respectively by *E. coli* and *Bacillus subtilis* are −47 mV and −33 mV.

**Figure 9:**
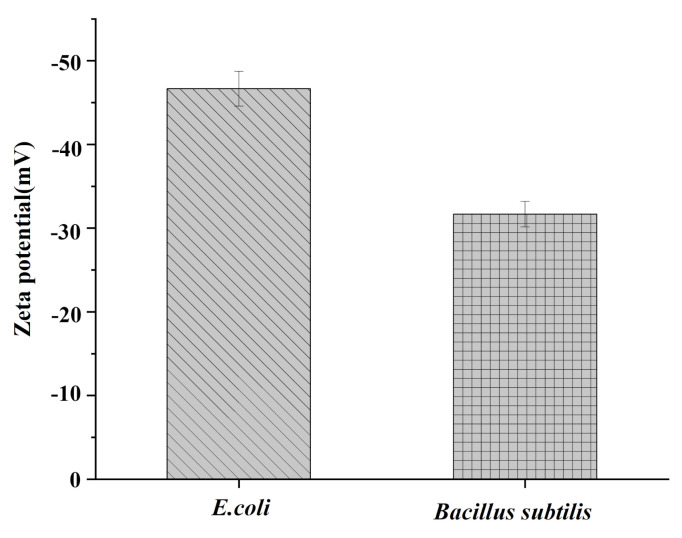
Zeta potential of *E. coli* and *Bacillus subtilis*. (pH = 7.4, 25 °C, 3 × 10^3^ CFU mL^−1^).

Figure 9 shows the Zeta potential of the two strains in PBS (0.01 mol·L^−1^, pH 7.4). At neutral pH, both strains had negative charges. Fe_3_O_4_@PDA@PEI MBs carry positive charges in the wide pH range of 2–11. Fe_3_O_4_@PDA@PEI MBs with positive charge can promote the interaction with negatively charged bacteria on the surface through electrostatic adsorption.

The effects of the amount and time of magnetic particles on the binding efficiency were preliminarily discussed. The supernatant of the newly cultured bacteria was centrifuged, and the supernatant medium was resuspended with 0.01 mol·L^−1^, pH 7.4 sterile PBS buffer, and rinsed three times. The PBS buffer was diluted to 10^3^ CFU·mL^−1^ level, and different amounts of MBs were added to the diluted bacterial solution according to different proportions. In order to combine the MBs, bacteria were incubated at constant temperature and constant speed for a certain time, and then the MBs-bacterial complex was magnetically separated. The supernatant was coated with plate culture medium, and the diluted bacterial solution, which was not combined with MBs was coated with plate culture. After the culture was completed, the capture efficiency was calculated. Capture efficiency (*CE*) of bacteria is determined by the following equation:

(2) $$CE=\left[\frac{N_{e}}{\left(N_{u}+N_{e}\right)}\right]\times 100\%.$$

*N_u_* is the number of uncombined bacteria (CFU mL^−1^), and *N_e_* is the number of bacteria enriched with MBs (CFU mL^−1^).

**Figure 10:**
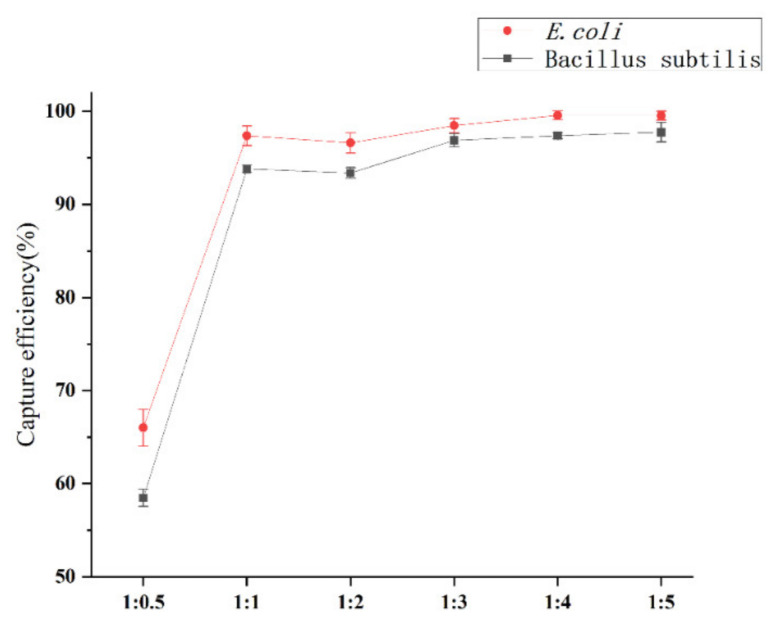
Capture Efficiency of Different Proportions of Fe_3_O_4_@PDA@PEI MBs (1 mg·mL^−1^) and Bacteria (3 × 10^3^ CFU mL^−1^) (*v*/*v*). (pH = 7.4, 25 °C).

It can be seen from Figure 10 that the capture rates of *Bacillus subtilis* and *E. coli* by Fe_3_O_4_@PDA@PEI MBs were 94.37% ± 0.78% and 99.54% ± 0.33%, respectively, when the number of bacteria was 1:1 and the capture time was as short as 5 min. With the increase of capture time, the capture rate remained stable. The magnetic beads have good capture efficiency for *E. coli*, and the capture efficiency is close to 100%, which means that almost all *E. coli* can be captured in the PBS system. For other enrichment of bacterial cells with magnetic procedures, direct enrichment of bacteria using bare magnetic beads takes up to 60 min but capture efficiency is only about 70% [43], and the capture efficiency of Fe_3_O_4_@Chitosan to these two kinds of bacteria is not higher than 75% [42].

**Figure 11:**
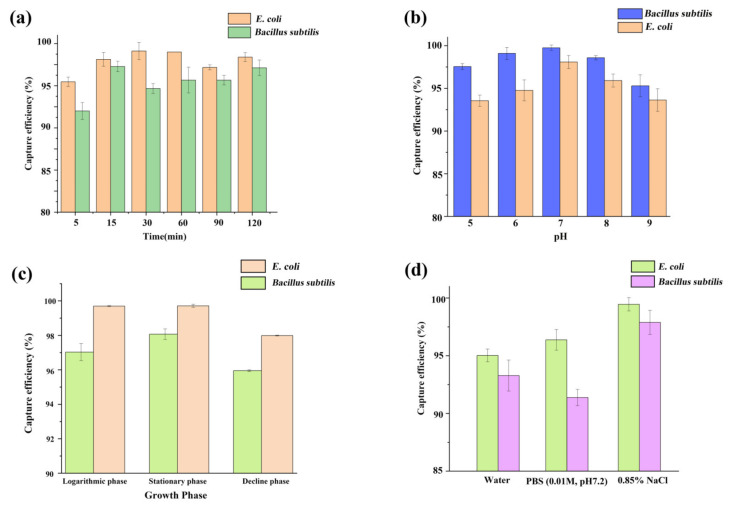
Effects of (**a**) incubation time, (**b**) pH, (**c**) growth phases, and (**d**) salinity on the capture efficiency.

Figure 11a demonstrates that no matter how much Fe_3_O_4_@PDA@PEI MBs and the capture time increase, the capture efficiency roughly reaches the peak within 15 min, and the capture efficiency does not change significantly with the increase of the amount of Fe_3_O_4_@PDA@PEI MBs. Therefore, when the bacterial concentration was 10^3^ CFU·mL^−1^, the volume ratio of MBs to the bacterial solution was 1:1, and the action time was 15 min, the maximum adsorption efficiency could be obtained.

#### 3.2.3. Effect of Buffer Environment on Binding

Based on the commonly used 0.01 M PBS buffer system, the effects of pH and concentration of buffer solution on the interaction between Fe_3_O_4_@PDA@PEI MBs and bacteria were tested. The pH of PBS buffer was adjusted to 5, 6, 7, 8, 9, respectively. After cleaning the fresh bacterial liquid, the PBS buffer was diluted to 10^3^ CFU·mL^−1^ level, and then combined with a certain amount of MBs, respectively. The binding efficiency was calculated according to the above method, as shown in Figure 11b.

It can be seen from Figure 11b that Fe_3_O_4_@PDA@PEI MBs had no significant difference in the capture rate of *Bacillus subtilis* and *E. coli* when the buffer pH was in the range of 5–9, and the capture rate was higher than 93%. The optimum pH range for the adsorption and capture of *Bacillus subtilis* and *E. coli* by Fe_3_O_4_@PDA@PEI MBs covered 5–9, and the near-neutrality was the best, almost 100%.

#### 3.2.4. Effect of Bacterial Activity on Binding

Considering the four stages of growth and reproduction of bacterial populations, the *E. coli* and *Bacillus subtilis* bacteria solution at logarithmic phase, stationary phase, and decline phase were respectively centrifuged and washed three times, and then diluted with 0.01 mol·L^−1^ (pH 7.2) sterile PBS buffer to 10^2^–10^4^ CFU·mL^−1^ level. A certain amount of MBs were added to the diluted bacteria liquid and incubated. After the MBs were fully combined with the bacteria, the MBs-bacterial complexes were separated under the assistance of a magnetic field, and the supernatant was coated with a plate culture medium. At the same time, the diluted bacterial liquid, which was not combined with MBs, was cultured on the plate. Then the capture efficiency was calculated.

As shown in Figure 11c, Fe_3_O_4_@PDA@PEI MBs had high capture efficiency for three kinds of bacteria at different growth phases in a slightly acidic to weakly alkaline buffer environment. The efficiency of *E. coli* and *Bacillus subtilis* were above 98.6% and 96%, respectively. There was no significant difference in binding and trapping efficiency due to the different growth phases of bacteria. 

Considering that some bacteria can produce spores to survive the adverse environment, Bacillus spores like Bacillus anthracis are also important biological warfare agents, which can be intentionally released, especially in the form of aerosols to pollute the air, water, and food in large areas. This study investigated the adsorption and capture effect of Fe_3_O_4_@PDA@PEI MBs on *Bacillus subtilis* spores. Table 1 displays that the adsorption efficiency was more than 99%. The results showed that the prepared Fe_3_O_4_@PDA@PEI MBs not only had high adsorption capture efficiency for bacterial propagules but also could efficiently capture *Bacillus subtilis* spores, which further verified the broad-spectrum bacterial capture ability of Fe_3_O_4_@PDA@PEI MBs.

#### 3.2.5. Effect of Ionic Strength on Binding

Based on the effect of buffer environment on the interaction between Fe_3_O_4_@PDA@PEI MBs and bacteria, the effects of common sampling liquids such as water, PBS (0.01 M, pH 7.2), and 0.85% NaCl on the interaction between MBs and bacteria were investigated. As shown in Figure 11d, water, PBS (0.01 M, pH 7.2), and 0.85% NaCl had little effect on the binding of Fe_3_O_4_@PDA@PEI MBs to bacteria, and the capture efficiency was nearest 100%.

### 3.3. Isolation and Enrichment Efficiency of Bacteria by Fe_3_O_4_@PDA@PEI MBs Verified by ATP Bioluminescence Detection

The effect of MBs enrichment combined with ATP-BL detection was preliminarily verified. A total of 50 μL of 6 × 10^2^ CFU mL^−1^ of *Bacillus subtilis* and 4 × 10^2^ CFU mL^−1^ of *E. coli* were taken for ATP-BL detection for three times. Then, 500 μL of the bacterial solution was added to 500 μL of MBs for enrichment. After magnetic separation, the supernatant was discarded and suspended in 50 μL of 0.01 mol L^−1^ sterile PBS buffer for ATP-BL detection. The results shown in Table 2 demonstrate that the enrichment of MBs could significantly enhance the ATP fluorescence detection effect.

## 4. Conclusions

In this paper, Fe_3_O_4_@PDA@PEI MBs with high-density positive charge were prepared as an adsorbent material. DA was self-polymerized on iron oxide to form PDA and then crosslinked PEI on the outermost layer of the magnetic beads to form Fe_3_O_4_@PDA@PEI MBs. The prepared Fe_3_O_4_@PDA@PEI MBs were then successfully used to concentrate low-level microorganisms in the liquid phase. This study demonstrated that Fe_3_O_4_@PDA@PEI MBs could interact directly with bacteria, and a large number of positive charges introduced by PEI play an important role in the interaction. The interaction between them is mainly based on electrostatic adsorption, and the components with negative charges on the surface of bacteria, such as LPS, proteins, peptidoglycans, and teichoic acid are critical for the broad-spectrum separation and enrichment of microorganisms in the liquid phase.

A sufficient amount of Fe_3_O_4_@PDA@PEI MBs have higher capture efficiency and faster capture speed for bacteria, most of which can reach nearly 100% adsorption capture efficiency within 3 min. Meanwhile, they are generally not affected by the adsorption solution, such as pH 5–8 or the different growth states of bacteria.

Fe_3_O_4_@PDA@PEI MBs can achieve rapid and broad-spectrum enrichment of bacteria. The results show that different bacteria can be efficiently captured and segregated by Fe_3_O_4_@PDA@PEI MBs under the external magnetic field, which will allow rapid determination of microbial concentration in water on site. Only the samples are collected into the liquid, Fe_3_O_4_@PDA@PEI MBs can be applied for bacteria enrichment. Compared with the MBs of other non-specific adsorption bacteria, PEI with the highest cationic density was selected as the adsorption layer, so that the charge on the surface of the Fe_3_O_4_@PDA@PEI MBs was up to +40 mV. Therefore, the adsorption efficiency of negative Gram-negative bacteria and Gram-positive bacteria in a neutral environment was extremely high. Compared with the harsh preparation conditions and preparation steps of other MBs [43,44], the preparation steps of this method were simple, and the preparation of beads could be completed at room temperature. Fe_3_O_4_@PDA@PEI MBs is a rapid, highly sensitive, and low-cost detection method, and has broad application prospects in the fields of enrichment and separation of environmental microorganisms and biological sensing detection.

## Figures and Tables

**Table 1:** Magnetic capture efficiency of Fe_3_O_4_@PDA@PEI MBs for *Bacillus subtilis*.

*Bacillus subtilis* Spores		
Original solution (CFU/100 μL)	5.46 × 10^4^	5.46 × 10^3^
After adsorption (CFU/100 μL)	282	34
Capture efficiency	99.49%	99.32%

**Table 2:** Magnetic capture efficiency of Fe_3_O_4_@PDA@PEI MBs on *Bacillus subtilis* and *E. coli*.

*E. coli*	Before/RLU	22 ± 1	20 ± 2	18 ± 3
	After/RLU	998 ± 40	1062 ± 51	1043 ± 70
*Bacillus subtilis*	Before/RLU	19 ± 6	14 ± 4	26 ± 4
	After/RLU	884 ± 11	1016 ± 22	1002 ± 30

## Data Availability

The data presented in this study are available upon request from the corresponding author.
